# Laparoscopic extraction of gastric self-expandable metallic stent after migration in ileum: A case report

**DOI:** 10.1016/j.ijscr.2018.10.041

**Published:** 2018-10-29

**Authors:** F.-X. Terryn, E. Dereeper, S. Lo Bue

**Affiliations:** a Department of General Surgery, Hôpital de Nivelles, Nivelles, Belgium; b Department of Anesthesiology, Hôpital de Nivelles, Nivelles, Belgium

**Keywords:** Laparoscopy, Self-expandable metallic stent (SEMS), Stent migration, Gastric banding, Sleeve gastrectomy, Case report

## Abstract

Revisional surgery in bariatric patients can sometimes lead to life-threatening complications that need a fast diagnosis and treatment as well as a multidisciplinary approach. If left undiagnosed or untreated, this may lead to sepsis, multiple organ failure, and death.

In this case report, we describe the management of a gastric perforation which occurred after conversion of a gastric banding to a sleeve gastrectomy.

## Highlights

• Revisional surgery in bariatric patients can sometimes lead to life-threatening complications.
• A good combination between endoscopic procedure and surgery is needed to treat a gastric perforation.
• Self-expandable stents have a high migration rate, and a laparoscopic extraction is feasible.

## 1 Introduction

Revisional surgery in bariatric patients can sometimes lead to life-threatening complications that need a fast diagnosis and treatment as well as a multidisciplinary approach. If left undiagnosed or untreated, this may lead to sepsis, multiple organ failure, and death.

In this case report, we describe the management of a gastric perforation which occured after conversion of a gastric banding to a sleeve gastrectomy.

This work has been reported in line with the SCARE criteria [1].

## 2 Presentation of case

A 38-year-old woman has a history of adjustable gastric banding in 2014 for morbid obesity. Because of an intragastric migration, this banding was removed endoscopically in october 2017. As the patient did not lose weight, we performed a laparoscopic sleeve gastrectomy in january 2018 after a multidisciplinary discussion who approved the surgery. Weight was 97 kg and BMI was 38,4 kg/m^2^.

**Fig. 1:**
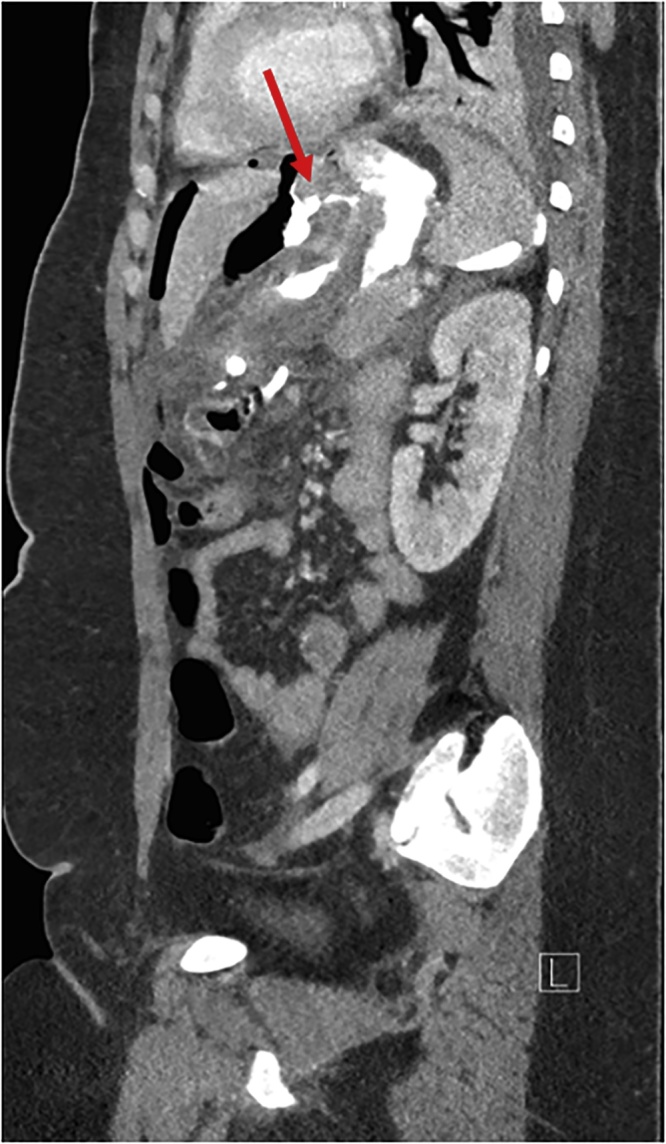
One day after laparoscopic gastric banding conversion to sleeve gastrectomy: sagittal plane of CT scan with obvious anterior cardial leak (red arrow).

The day after the surgery, there was a clinical suspicion of peritonitis. A CT-scan (Fig. 1) confirmed this diagnosis and suggested a gastrojejunal leak. We performed a laparoscopic suture of an anterior cardial perforation on the previous gastric banding position, omentoplasty, lavage and drainage. There was no insufficiency on the staples lines.

The perforation did not heal after this second operation and five days later, the patient required two complementary percutaneous pleural and abdominal drainages and a 2 days stay in our intensive care unit for a sepsis management (Dindo-Clavien 4b).

**Fig. 2:**
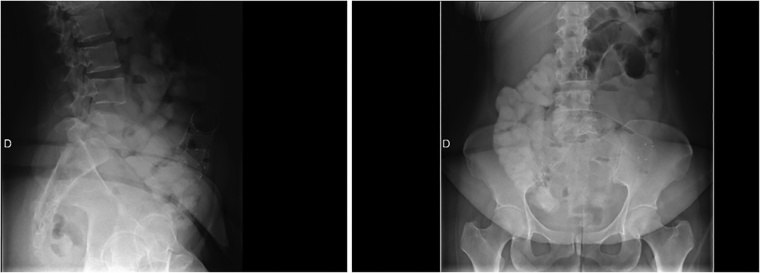
Radiographs of the migrated self-expandable gastric stent.

After recovery, on the third postoperative week, we decided to perform a gastric self-expandable stent implantation to cover the perforation. Unfortunately, this prosthesis migrated two weeks after its implantation to the small intestine. An endoscopic extraction was not possible because of its ileal location (Fig. 2). Nevertheless, the gastroscopy confirmed the healing of the gastric perforation, so the patient was allowed to resume enteral feeding.

**Fig. 3:**
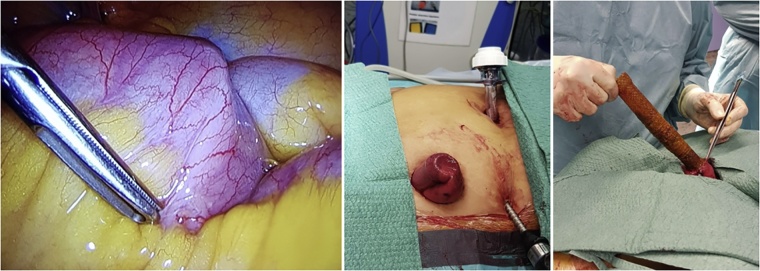
Laparoscopic removal of self-expandable gastric stent.

Thus, we performed a laparoscopic extraction of the prosthesis six weeks after the sleeve gastrectomy (Fig. 3). No complication followed this last surgery.

Two months postoperatively, the patient had lost 13 kg (total body weight loss was 13,4%, BMI is now 33 kg/m^2^, and %EBMIL is 40%).

## 3 Discussion

As we mentionned, there was no insufficiency on the staples lines to be seen during the laparoscopic exploration and the leak was likely to be related to the severe adhesional status between the liver and stomach caused by the intragastric migration of the gastric banding.

Laparoscopic conversion of gastric banding to sleeve gastrectomy following poor weight loss results is a common procedure in our institution. According to Janik et al. and Spaniolas et al., the conversion from laparoscopic banding to sleeve gastrectomy leads to lesser morbidity than to laparoscopic Roux-en-Y gastric bypass [2,3].

Because the patient showed early signs of peritonitis, we preferred the surgical option at first. There was only a 24 h delay between the laparoscopic conversion to sleeve gastrectomy and the laparoscopic suture of the gastric perforation.

Okazaki et al. recently showed that endoscopic treatment of gastric fistulas seems to be a safe and effective procedure on selected patients [4]. Stent implacement is also recommended by the American Society for Metabolic and Bariatric Surgery to treat fistulas developing after bariatric surgery [5]. In this case, we chose a self-expandable gastric stent instead of good endoscopic alternative procedures such as endoscopic vacuum assisted closure therapy. Of course this could have been a good alternative.

Intestinal bleeding or aorto-oesophageal fistulas as described complications after self-expandable metal stent implantation but the most frequent is the stent migration with an approximate rate of 20–40% [6]. Unfortunately, a retrospective review of Singer et al. found no pattern to reduce it [7].

## 4 Conclusion

In the future, we will likely observe an increase in gastric banding conversion to other bariatric surgical procedures for patients with poor results on excess weight loss. Life-threatening complications such as leaks can occur. For non-conservative cases, a good combination of surgical and endoscopic procedures and a multidisciplinary approach are needed to resolve the problem successfully. Of course, a bigger number of cases would be necessary to prove and deepen these results.

## Conflicts of interest

All authors declare to have no conflicts of interest.

## Sources of funding

All authors declare to have no source of fundings.

## Ethical approval

The study is exempt from ethnical approval in our institution.

## Consent

We obtained the patient’s consent and added a consent section in the manuscript.

## Author contributions

All authors were in charge of the patient and contributed equally in the realisation of this manuscript.

Salvatore Lo Bue is the head surgeon and performed the operation, I (François-Xavier Terryn) am the trainee surgeon and assisted the intervention. Etienne Dereeper is the anesthesiologist who took care of the patient.

I was in charge of the redaction of the case and both of the co-authors made interesting comments on my first version of this case report, especially about the discussion and helped with the review of the literature.

## Registration of research studies

Not applicable for this case report.

## Guarantor

François-Xavier Terryn.

## Provenance and peer review

Not commissioned, externally peer reviewed.
